# Balneotherapy Together with a Psychoeducation Program for Benzodiazepine Withdrawal: A Feasibility Study

**DOI:** 10.1155/2016/8961709

**Published:** 2016-11-13

**Authors:** P. De Maricourt, P. Gorwood, Th. Hergueta, A. Galinowski, R. Salamon, A. Diallo, C. Vaugeois, J. P. Lépine, J. P. Olié, O. Dubois

**Affiliations:** ^1^Service Hospitalo Universitaire de Santé Mentale et de Thérapeutique, Université Paris Descartes, Centre Hospitalier Sainte Anne, Paris, France; ^2^INSERM UMR 894, PRES Sorbonne Centre de Psychiatrie et Neurosciences, Université Paris Descartes, Sorbonne Paris Cité, Paris, France; ^3^Laboratoire de Psychopathologie et Processus de Santé (EA 4057), Institut de Psychologie, Université Paris Descartes, Sorbonne Paris Cité, Paris, France; ^4^Institute of Public Health, Epidemiology and Development “ISPED”, Bordeaux University, Bordeaux, France; ^5^Le Manoir Clinic, 09400 Ussat les Bains, France; ^6^APHP, Département de Psychiatrie et de Médecine Addictologique, Hôpital Fernand Vidal, Paris, France; ^7^Saujon Clinic, BP 30, 17600 Saujon, France

## Abstract

Benzodiazepines should be prescribed on a short-term basis, but a significant proportion of patients (%) use them for more than 6 months, constituting a serious public health issue. Indeed, few strategies are effective in helping patients to discontinue long-term benzodiazepine treatments. The aim of this study was to assess the feasibility and the impact of a program including cognitive behavioural therapy, psychoeducation, and balneotherapy in a spa resort to facilitate long-term discontinuation of benzodiazepines. We conducted a prospective multicentre cohort study. Patients with long-term benzodiazepine use were recruited with the aim of anxiolytic withdrawal by means of a psychoeducational program and daily balneotherapy during 3 weeks. The primary efficacy outcome measure was benzodiazepine use 6 months after the program, compared to use at baseline. A total of 70 subjects were enrolled. At 6 months, overall benzodiazepine intake had decreased by 75.3%, with 41.4% of patients completely stopping benzodiazepine use. The results also suggest a significantly greater improvement in anxiety and depression symptoms among patients who discontinued benzodiazepines compared to patients who only reduced their use. Our findings suggest that balneotherapy in association with a psychoeducative program is efficient in subjects with benzodiazepine addiction.

## 1. Introduction

Among psychotropic drugs, benzodiazepines are one of the most widely prescribed. Given their sedative, anxiolytic, muscle relaxant, anticonvulsive, and hypnotic properties, benzodiazepines are widely used in the short-term management of anxiety, insomnia, alcohol withdrawal, seizures, or agitation. Their long-term use is not recommended for several reasons including misuse with tolerance, dependence, decreased alertness, and the potential risk of domestic or traffic accidents. Furthermore, long-term use has been associated with cognitive impairment especially in elderly patients, with recent studies highlighting a possible link with dementia and particularly Alzheimer's disease [1].

Prevalence rates of long-term benzodiazepine use vary widely in older studies [2] with differing eligibility criteria. A study of the literature estimated the prevalence to be between 2.2 and 17.6% [3]. Results of the ESEMeD/MHEDEA 2000 epidemiological study showed that in France 21% of a sample of the general population (*n* = 580) had taken at least one psychotropic drug during the current year, an anxiolytic or hypnotic drug for 19% of subjects, often on a long-term basis (3 to 6 months: 6.7%; >6 months: 23%) [4, 5].

Reducing the consumption of benzodiazepines and promoting their proper use is a major public health issue. Thus seeking new strategies to help patients discontinue long-term benzodiazepine use is essential. Indeed while many strategies have been tried the rates of effective discontinuation remain low [6].

Balneotherapy (BT) with mineral water and other types of somatic care in the environment of a spa resort can be used for the treatment of affective disorders, anxiety disorders, and withdrawal syndromes.

Recently, BT has been shown to be effective in the treatment of generalized anxiety disorder (GAD) [7, 8]. A prospective, randomized, multicentric, controlled clinical trial demonstrated the significant superiority of BT over paroxetine treatment in terms of efficacy and tolerance. Furthermore, a pilot study has suggested that balneotherapy improves the psychological symptoms of occupational burnout [9].

Few studies have attempted to explain the pathophysiological mechanisms underlying the effectiveness of balneotherapy. However, a decrease in salivary cortisol levels, a stress marker that is modified by psychotropic drugs, was reported after spa bathing [10] and the affinity of the serotonin transporter, altered in depression, appears to be increased between 30 min and 1 week after balneotherapy in ozonized water [11]. Warm footbaths have been shown to induce relaxation with a concomitant decrease in sympathetic tone and serum cortisol levels as well as an elevation in salivary secretory IgA titers [12]. A local effect through nociceptive skin receptors and central effects on endorphins and immune factors have been suggested by Lange et al. [13]. Moreover, balneotherapy by fibromyalgia patients stimulated the hypothalamic axis, corticotropic releasing factor, and adrenocorticotropic hormone in correlation with pain decrease and improvements in depression and quality of life [14]. Another hypothesis is that balneotherapy could stimulate *α*MSH cutaneous cells which could activate the corticotropic system [15]. Lastly, gate control theory explains why heat and the hydrostatic pressure of water on the body surface can decrease the sensation of pain [16].

Psychoeducation is a psychosocial intervention centred on cognitive and behavioral therapy.

The aim of this approach is to teach patients to understand their illness and its treatment, to manage everyday symptoms, and to improve coping strategies.

A large number of studies have shown this strategy to be effective in major depressive disorders (in particular relapse/recurrence prevention) [17, 18], bipolar disorders [19, 20], and schizophrenia [21] as well as for addiction [22] and anxiety disorder [23, 24].

The present study was performed in anxious outpatients with long-term (more than 6 months) benzodiazepine use. The aim was to study the interest of a psychoeducation program together with balneotherapy in a spa resort for benzodiazepine withdrawal and the feasibility of such a program. We expected a significant decrease in benzodiazepine intake following the program.

## 2. Methods

### 2.1. Study Design and Patients

**Figure 1:**
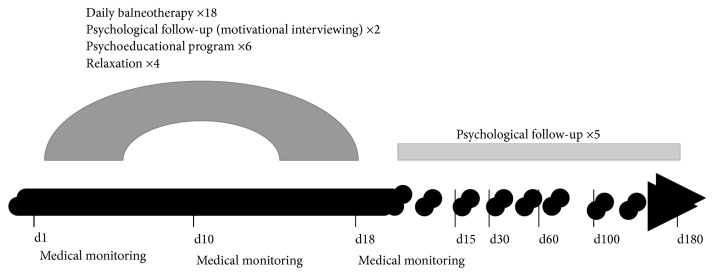
Study design.

The SPECTh (Sevrage de Psychotropes par Education Psychothérapeutique en Cure Thermale) study was a prospective multicentre cohort study conducted in 4 spa resorts in France that offer balneotherapy for psychosomatic disorders (Bagnères de Bigorre, Néris les Bains, Saujon, and Ussat les Bains) between April 2010 and November 2011 (Figure 1).

Inclusion criteria for patients recruited by a committee of spa doctors in each one of the 4 centres were

- age between 18 and 85 years,
- DSM IV TR (American Psychiatric Association, Diagnostic and Statistical Manual of Mental Disorders, 4th Edition, Text Revision) criteria for benzodiazepine abuse or dependence lasting more than 6 months,
- a history of withdrawal failure,
- motivation for stopping benzodiazepine use,
- no need for hospitalization.

Patients with alcohol or other substance abuse/dependence, psychosis, borderline, and antisocial personality disorders were not eligible using DSM IV TR criteria.

The following substances were considered as benzodiazepines or equivalent: alprazolam, bromazepam, prazepam, lorazepam, clonazepam, oxazepam, diazepam, tetrazepam, clorazepate dipotassium, clotiazepam, nordazepam, and hypnotics: zolpidem, lormetazepam, zopiclone, loprazolam, and temazepam. All participants gave their written consent prior to study enrolment and could drop-out of the study at any time.

The protocol was designed by a panel of French experts and based on current clinical practice. It included the following:

- Medical monitoring, to assess benzodiazepine withdrawal in particular
- Daily balneotherapy during 3 weeks using natural spring mineral water including. Each day, participants had 4 care sessions during 90 minutes for a total of 72 sessions during the program. These cares were as follows:
  - Bubbling bath (10 min)
  - Balneotherapy shower with a mean pressure of 3.5 bar/mn and temperature at 37-38°C, targeting the abdominal, paravertebral, and cervicobrachial regions
  - Underwater massages by experienced physiotherapists (10 min)
  - Bath in a swimming pool of mineral water (10 mn) with a temperature at 31-32°C
- A psychoeducation program within the framework of a cognitive-behavioral approach in groups of 8 to 12 patients, for 6 sessions lasting 1:30 to 2:00 hours. Through this, patients should be empowered to understand the illness and cope with it in a successful manner. Each session focused on a specific topic addressed in the following order:
  - Session number 1: the mechanisms of addiction, benzodiazepine's characteristics
  - Session number 2: stopping benzodiazepines and the process of recovery, Prochaska and DiClemente's Transtheoretical Model of Change
  - Session number 3: understanding anxiety and stress
  - Session number 4: description of the disorders associated with stress and anxiety
  - Session number 5: alternatives to benzodiazepine treatment (CBT, relaxation, nonaddictive anxiety medications, and so on)
  - Session number 6: introduction to methods to improve self-assertiveness
- Psychological consultations, which included an interview at the beginning of the intervention to assess and stimulate the patient's motivation, a second at the end of the program to set targets and assessments after discharge, and then consultations at 15, 30, 60, 100, and 180 days after the end of the program
- Four sessions of specialized relaxation. Relaxation training is a common treatment for anxiety disorders and studies show consistent and significant effectiveness of relaxation training in reducing anxiety [25]. The program consisted of training sessions with different relaxation methods: abdominal breathing, progressive muscle relaxation, and autogenic training

Instructions were prepared in order to homogenize the protocol in the 4 centres and to ensure program quality, reproducibility, and comparability. Training was provided to each psychologist who conducted the workshops.

### 2.2. Measures

The primary efficacy outcome measure was benzodiazepine intake at 3 and 6 months after the program was completed, compared to baseline.

The secondary outcome measures were

- benzodiazepine intake between consecutive assessment time points,
- anxiety and depression scores using the Hospital Anxiety and Depression Scale (HAD scale) [26] and the Beck Depression Inventory (BDI) [27] at baseline and at the last available assessment
- Level of addiction using a specific scale (the ECAB, the “cognitive attachment to benzodiazepines” scale) [28], between baseline and the last available assessment.
- Sleep quality at baseline, and at each consecutive assessment point on a scale ranging from 0 (excellent sleep) to 10 (poor sleep).

### 2.3. Statistical Analysis

Analyses were performed using SAS software (9.1.3, SAS Institute Inc., Cary, NC, USA). Values are expressed as means (standard deviation (SD)) or as percentages. Student *t*-test or chi-squared tests were used to compare differences between groups for numerical variables with normal distribution or the Wilcoxon signed-rank when the sample data are not normally distributed.

## 3. Results

Nine patient groups were formed: 1 in Bagnères de Bigorre with 8 patients, 2 in Néris les Bains with 13 patients, 4 in Saujon with a total of 39 patients, and 2 in Ussat les Bains with 10 patients. A total of 73 patients were included. Three patients dropped out before the beginning of BT and 4 patients dropped out after having started BT sessions. The drop-out group did not differ on demographic and clinical status variables. The reason for dropping out was a significant lack of motivation to stop benzodiazepines and continue the program despite psychological consultations at the beginning of the intervention to assess and stimulate the patient's motivation.

Women constituted 78.5% (55/70) of the sample. The average age was 54 years and 9 months (±standard deviation: 10 years and 4 months) and 80.3% of patients had been using benzodiazepines for more than 3 years (Table 1).

Results for the primary outcome measure show that, after 6 months, 41.4% (*n* = 29) of patients had completely stopped using benzodiazepines.

Overall, benzodiazepine intake was reduced by 75.3% in patients completing the 6-month assessment. Patients taking only one benzodiazepine drug at baseline stopped more frequently than others (*p* < 0.006). 17% of patients have no improvement at the end of 6 month follow-up (Data not shown). A descriptive analysis showed that this program seems to be more effective among men than with women and in patients with a professional activity in comparison to those who did not work. Scores for the secondary outcome measures are summarized in Table 2.

The change in mean ECAB scores between baseline and the 6-month follow-up was a significant decrease (−3.46, *p* < 10^−6^). The score was significantly lower in the group that totally stopped versus the group that continued using benzodiazepines (resp., −5.19 versus −2.14; *p* = 0.002).

After the program depressive and anxiety symptoms measured with the HAD scale and with the HAD anxiety and depression subscales were significantly improved (resp., −8.1, −4.66, and −3.2; with *p* = 0.001, *p* = 0.001, and *p* = 0.01). These differences were significantly larger in the group that completely stopped compared with the group that did not stop using benzodiazepines (*p* = 0.002).

While overall improvement measured with the Beck Depression Inventory was also significant at 6 months (−5.49, *p* = 0.01), the reduction in scores in the group that completely stopped using benzodiazepines compared with the group that continued using benzodiazepines did not reach significance (*p* = 0.11).

Concerning the assessment of sleep quality, scores were significantly lower after the program (−1.2, *p* = 0.009). This difference was particularly significant in the group that stopped using benzodiazepines (−2.19, *p* = 0.004) compared with the group of patients who continued to use them.

## 4. Discussion

To our knowledge, the present study is the first assessing the benefit of balneotherapy combined with a psychoeducational program for benzodiazepine withdrawal. Benzodiazepines show rapid and potent anxiolytic effects but have adverse side effects as well, namely, dependence, drowsiness, and impaired cognition and memory, and their excessive use constitutes a serious public health problem. Consequently, there is a need for alternative approaches targeting anxiety with nonpharmacological treatments. The combination of balneotherapy and cognitive and behavioral therapy techniques as performed here is interesting and relevant for benzodiazepine withdrawal programs.

Our findings indicate that this combination was effective, with a success rate of more than 41% (percentage of patients maintaining benzodiazepine abstinence for 3 and 6 months) and an overall decrease at 6 months of 75% of the benzodiazepine intake at baseline.

The results suggest that this original program could be an alternative strategy in the management of these patients.

We also found a significant improvement in depression and anxiety levels, particularly in the group that had totally stopped benzodiazepine intake at the 6-month follow-up evaluation. Furthermore, total discontinuation had a significant impact on sleep quality. Our data suggest that total discontinuation of benzodiazepine use is indeed a justified objective. As previously reported by our group [7], these results advocate the use of balneotherapy in patients with anxiety disorders. Nevertheless some limitations to the present study should be discussed.

### 4.1. Limitations

The first limitation is the lack of a control group and of randomization. Besides the fact that controlling for both balneotherapy and psychoeducation would have required a much larger sample, the aim of the study was to examine the feasibility of the program. Second, our study was limited to 6-month follow-up. It would be interesting to extend the duration of follow-up to at least one year since remitted anxiety recurs in 24% of patients within 2 years [29] and more often in women (64% within 3 years) [30]. Also, more specific questions about benzodiazepine use as well as urine benzodiazepine assays could have been informative.

Moreover, the study provided no information on other psychotropic medications and on comorbid mental disorders although both factors may interfere with benzodiazepine withdrawal [31].

Another limitation is the sample size (*n* = 70). Only highly motivated subjects referred by their general practitioner were enrolled. Spas are often located in small towns far from research centres. Moreover, the program required the patient to commit 3 whole weeks, a long period of constraint to the spa, limiting recruitment among subjects in active employment. Therefore, our results cannot be extrapolated to all patients with benzodiazepine addiction.

The protocol is ongoing in French spa resorts. This will allow new analyses on a larger database and a confirmatory controlled study but finding a suitable control condition is not easy.

### 4.2. Qualitative Aspects of the Study

This study was a feasibility study. Showing the effectiveness of the program was an important objective of this study but showing the feasibility of the protocol was also crucial. Indeed one of the objectives is to generalize this protocol in current practice. For that it was important to assess the feasibility of the program.

Two pieces of data can guide us. Patient compliance with a low rate of premature exits despite a demanding program (more than two absences led to exclusion of the program) and a low drop-out rate (4 patients dropped after having begun the protocol).

Patient satisfaction rate was 93% at the end of the program.

Indeed in terms of implementation, the low rate of drop-out and the level of satisfaction among patients support the feasibility of such a program despite its prerequisites (time constraint, specific setting in a spa resort, and risk of patient drop-out).

## 5. Conclusion

Despite its limitations, the present feasibility study suggests that a program of psychoeducation (including CBT) combined with balneotherapy for benzodiazepine withdrawal run in a spa resort is a promising strategy to stop long-term benzodiazepine use. We showed that this approach is feasible, effective, and a well-tolerated treatment for benzodiazepine addiction, a condition that currently lacks a standard treatment. Future clinical trials with a randomized controlled design are needed to confirm the reliability and validity of these encouraging results.

## Figures and Tables

**Table 1:** Baseline demographic and clinical characteristics of patients.

	*N*	%
Age, mean ± SD		
54,75, ± 10,4		
Sex (nb, %)		
Male	15	21,43
Female	55	78.57
Current marital status (nb, %)		
Married	41	58.57
Single	15	21.43
Divorced	11	15.71
Widowed	2	2.86
Cohabiting	1	1.43
Total	70	100.00
Current employment status (nb, %)		
Pensioner	23	33.82
Unemployed	20	29.41
Employee	19	27.94
Executive or liberal profession	5	7.35
Worker	1	1.47
Total	68	100.00
Benzodiazepine intake at baseline: number of different benzodiazepine molecules (no, %)		
1	29	41.43
2	26	37.14
3	13	18.57
5	1	1.43
6	1	1.43
Benzodiazepine equivalent dose (diazepam), mean ± SD		
15.3 mg ± 12.4		
Duration of benzodiazepine use before inclusion (nb, %)		
≥3 years	53	80.30
≥1 years	6	9.09
≥6 months	5	7.58
between 3 and 6 months	2	3.03

**Table 2:** Difference between baseline and 6-month follow-up scores of patients who succeeded, or not, in stopping benzodiazepines.

		Stopped benzodiazepine use				Stopped benzodiazepine use		
		1: yes	0: no	Total		1: yes	0: no	Total
		Baseline				End of study (6 months)		
ECAB	*N*	29	41	70		25	33	58
	M	6.75	6.92	6.85		1.56	4.78	3.39
	SD	1.58	1.87	1.75		1.21	2.82	2.71

*Comparison, baseline versus 6 months*								*p* < 10^−6^

*Comparison, “yes” versus “no”*				*p* = 0.69				*p* < 10^−4^

HAD anxiety	*N*	29	41	70		27	32	59
	M	14.31	12.8	13.42		7.37	9.93	8.76
	SD	4.39	4.25	4.36		3.54	5.02	4.57

*Comparison, baseline versus 6 months*								*p* = 0.001

*Comparison, “yes” versus “no”*				*p* = 0.15				*p* = 0.02

HAD depression	*N*	29	41	70		27	32	59
	M	9.41	8	8.58		4.07	6.5	5.38
	SD	4.85	4.42	4.64		3.56	4.77	4.41

*Comparison, baseline versus 6 months*								*p* = 0.01

*Comparison, “yes” versus “no”*				*p* = 0.21				*p* = 0.35

HAD total	*N*	29	41	70		27	32	59
	M	23.72	20.80	22.01		11.44	15.93	13.91
	SD	7.77	7.42	7.67		6.41	9.53	8.54

*Comparison, baseline versus 6 months*								*p* = 0.001

*Comparison, “yes” versus “no”*				*p* = 0.11				*p* = 0.02

Beck	*N*	29	41	70		27	32	59
	M	14.89	11.07	12.65		5.66	9.03	7.49
	SD	8.07	8.27	8.37		7.42	8.25	8.03

*Comparison, baseline versus 6 months*						*p* < 10^−6^		*p* = 0.01

*Comparison, “yes” versus “no”*				*p* = 0.06				*p* = 0.11

Quality of sleep	*N*	29	40	69		24	31	55
	M	5.62	6.12	5.91		4	5.41	4.8
	SD	2.44	1.95	2.17		2.52	2.79	2.75

*Comparison, baseline versus 6 months*						*p* = 0.004	*p* = 0.35	*p* = 0.009

*Comparison, “yes” versus “no”*				*p* = 0.34				*p* = 0.05

*N*: number, M: mean, and SD: standard deviation.
